# School-based surveillance on visit-to-visit blood pressure variability and high blood pressure in children and adolescents

**DOI:** 10.1186/s12872-021-01947-1

**Published:** 2021-03-17

**Authors:** Jiaxiang Wang, Hui Shen, Jieyu Liu, Chengqi Xiao, Cailong Chen, Haoyue Teng, Jia Hu, Jieyun Yin

**Affiliations:** 1 Jiangsu Key Laboratory of Preventive and Translational Medicine for Geriatric Diseases, School of Public Health, Medical College of Soochow University, 199 Renai Road, Suzhou, 215123 Jiangsu China; 2 Suzhou Center for Disease Prevention and Control, 72 Sanxiang Road, Suzhou, 215004 Jiangsu China; 3 Children Health Management Center, Children’s Hospital of Soochow University, Suzhou, Jiangsu China

**Keywords:** Visit-to-visit blood pressure variability, Pediatrics, High blood pressure, China

## Abstract

**Background:**

The predictive importance of visit-to-visit blood pressure variability (VVV) for high blood pressure (HBP) in a pediatric population has been largely unsettled. We aimed to evaluate it based on Health Promotion Program for Children and Adolescents (HPPCA), a school-based surveillance conducted from 2012 to 2018 in Suzhou, China.

**Methods:**

A total of 330,618 participants had BP measurement in 2018 and ≥ 3 BP records during 2012–2017, were recruited from HPPCA. Absolute BP values (in mmHg) were converted into age-, sex- and height- normalized z-scores. VVV was expressed as standard deviation (SD), coefficient of variation (CV) or average real variability (ARV) of BP z-scores during 2012–2017. Logistic regression models were used to assess the associations between VVV and HBP in 2018.

**Results:**

In 2018, 42,554 (12.87%) subjects were defined as HBP. VVV, except for SBP-CV and DBP-CV, was significantly higher in the HBP group than normotensives group. After adjusting for covariates including mean BP values from 2012 to 2017, SBP-SD, SBP-ARV, DBP-SD and DBP-ARV, increased the risk of HBP by 5.70 [95% confidence interval (95% CI) 5.54–5.87], 4.10 (95% CI 4.01–4.20), 4.70 (95% CI 4.50–4.90) and 3.39 (95% CI 3.28–3.50) times, respectively. Notably, SBP-SD significantly improved risk discrimination of HBP based on other risk variables (*c*-statistics, net reclassification index and integrated discrimination improvement significantly increased).

**Conclusions:**

Higher SD or ARV of BP, was independently related with higher probability of HBP in Chinese pediatric population. SBP-SD could be potentially helpful for detecting HBP. Future researches investigating the predictive value of VVV are warrant.

**Supplementary Information:**

The online version contains supplementary material available at 10.1186/s12872-021-01947-1.

## Background

High blood pressure (HBP) or hypertension is a serious public health problem worldwide [1], especially in China that possesses the largest population [2]. In recent decades, the incidence of HBP or hypertension is rising in children and adolescents [3]. It is well acknowledged that the natural history of essential hypertension origins from childhood [4]. Meanwhile, children and adolescents with high BP are more likely to develop cardiovascular disease (CVD) [5], cognition impairment [6], and eventually death [7] later in life.

BP is one of the most dynamic physiologic variables [8]. BP variability (BPV) can be accessed as variability of beat to beat (very short-term BPV), over 24 h (short-term BPV), day-to-day (midterm BPV), and from visit-to-visit (long-term BPV) [8]. As a typical type of BPV, visit-to-visit BP variability (VVV) was demonstrated not to be random, but significantly reproducible over a long period of follow-up [9].

In adults, ample evidence indicated that higher VVV was associated with the development, progression and severity of vascular, cardiac and renal target-organ damage, and with an increased risk of cardiovascular events and cardiovascular and all-cause mortality, independent of average BP values [10–12]. In addition, higher VVV and impaired cognitive function was observed in the elderly [13, 14]. Studies also suggested that, VVV was promising in facilitating risk classification for further cardiovascular disease [15].

While BPV has been relatively clearly understood in the adult literature, the significance of BPV in children and adolescents is not well described. In different pediatric disease settings, 24 h BPV has been tied to hypertension [16], left ventricular mass index [17], and arterial stiffness [18]. The 24-h BPV needs ambulatory BP monitoring, which is rather problematic to be interpreted in very young children [19]; while VVV can be determined relatively conveniently in clinical setting [20]. In children and young adults, increased VVV was shown to predict adult hypertension [21]. Also, higher systolic VVV was independently related to impaired neurocognitive function [22] in pediatric renal disease patients. What’s more, it has been reported that in children with primary proteinuric glomerulopathies, greater VVV was an independent predictor of poor clinical outcomes (e.g., end-stage renal disease or estimated glomerular filtration rate decline) [23]. However, the literature with longitudinal studies linking VVV to future HBP among general childhood and adolescence is rather scarce.

Utilizing data of Suzhou Health Promotion Program for Children and Adolescents (HPPCA), the present study aimed to examine the associations between VVV and risk of HBP, and investigate whether any VVV indices was capable of improving risk discrimination for HBP in a large-scale Chinese pediatric population.

## Methods

### Study area and population

**Fig. 1:**
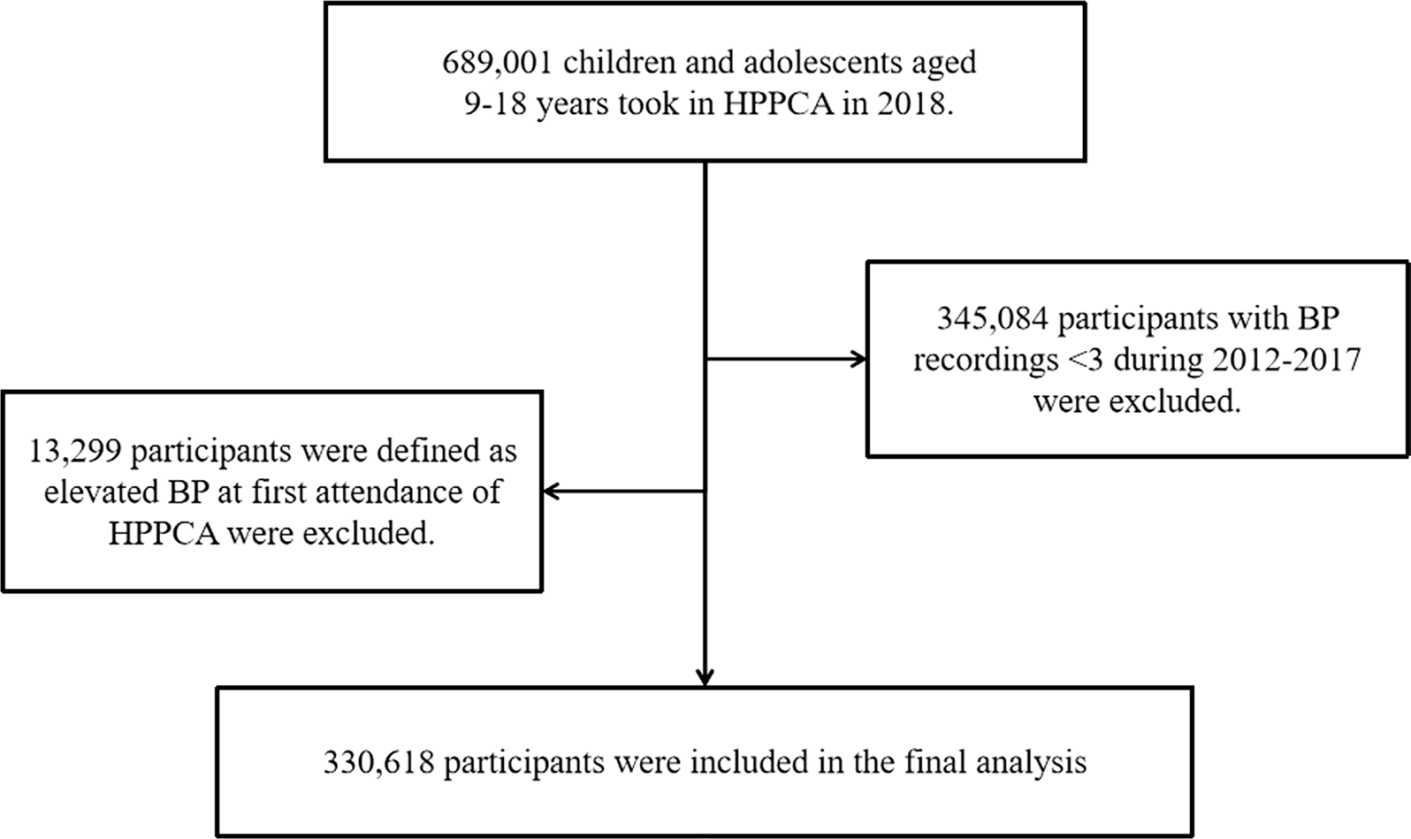
Flow diagram of study population selection

This study was a retrospective review chart that used data of seven consecutive years (from 2012 to 2018) from HPPCA. Detailed descriptions of HPPCA were reported previously [24]. In brief, aimed to promote a healthy lifestyle among children and adolescents, HPPCA was designed to annually assess the health status (i.e., BP and BMI) of all potential students aged 6–18 years from primary and secondary schools in Suzhou city. In this study, in order to have enough time span to trace historical data of participants, a total of 689,001 children and adolescents aged 9–18 years who took in HPPCA in 2018 were enrolled. After excluding participants with BP recordings < 3 during 2012–2017 (n = 345,084) and who were defined as HBP at first attendance of HPPCA (n = 13,299), a total of 330,618 participants were eventually included in the analysis. Figure 1 shows the details of the study population selection.

For the recruited participants, informed consent forms in writing duly signed by their guardians were collected before their examination. This study was approved by the Ethics Committee of Soochow University and Suzhou Center for Disease Prevention and Control.

### Covariates

When children and adolescents took physical examination in HPPCA, information including age, sex, weight, height, body-mass index (BMI), BP, region, and socioeconomic status (SES) were collected. Weight and height were measured with children in light clothing and without shoes. BMI was calculated as weight in kilograms divided by square of height in meters (kg/m^2^). BMI was transformed into a z-score corresponding to the age- and sex-specific reference outlined by the World Health Organization (WHO) [25]. Overweight and obesity was defined according to the WHO Child Growth Standards [25].

### BP measurements and definition

Systolic BP (SBP) and diastolic BP (DBP) were measured using validated oscillometric devices [26] (OMRON HEM752, HBP-1300, et al., available site: http://www.dableducational.org/index.html) after each subject had rested for at least 15 min in a sitting position. If the averaged oscillometric reading was at or above 90th percentile for age, sex, and height, BP was measured using an auscultation mercury sphygmomanometer with an appropriate cuff size for children [27]. SBP was defined as the onset of ‘tapping’ Korotkoff sounds, and DBP was defined as the fifth Korotkoff sounds [28]. The average value of three readings at a single visit was calculated and recorded for each child. All measurements were performed by well-trained health professionals using the same type of apparatus and following the same procedures.

Due to the age-related changes in body size/height, there was a need to convert casual BP values (in mmHg) into age-, sex- and height- normalized z-scores in order to compare a child's BP with the BP of healthy children at the same age, but also of children at different ages [29]. To test whether the school-based study was representative of the entire school population, we converted all the BP data into z-scores. The 330,618 subjects in the present study were representative of the entire population because their z-scores in 2018 (0.18 for SBP, 0.45 for DBP, 0.01 for height, and -0.01 for weight) were not significantly different than zero (*P* > 0.05).

In the current study, the average of three separate BPs at a single visit in 2018 was adopted to determine the status of HBP or high normal blood pressure (HNBP). HBP in youth was defined as SBP or DBP at least 95th age-, sex- and height-specific percentile according to the national BP reference for Chinese children considering influence of height, national blood pressure reference for Chinese Han children and adolescents aged 7 to 17 years (CCBP) [30]. Children with average SBP or DBP ≥ the 90th percentile but less than the 95th percentile, or adolescents with BP levels at 120/80 mmHg or above (but < 95th percentile), were considered HNBP [30].

### Assessment of VVV

To acquire VVV, at least three times of BP measurement before 2018 were obtained from each child and adolescent. VVV during 2012 and 2017 was assessed by three methods: (1) standard deviation (SD), (2) coefficient of variation (CV), and (3) average real variability (ARV), with all BPs indexed to z-scores [29]. The ARV method quantifies variability between adjacent readings and is defined by the following formula*:*

$${ARV}_{BP}=\frac{1}{n-1}{\sum }_{i=1}^{n-1}\left|{BP}_{i+1}-{BP}_{i}\right|.$$

### Statistical analysis

Continuous and categorical variables were presented as mean ± standard deviation (SD) and frequency (percentage), respectively. Characteristics of children or adolescents who developed HNBP or HBP versus those who remained normotensive were compared using 1-way Analysis of Variance (ANOVA), Wilcoxon ranked sum test or Chi-square test, when appropriate. Both BP levels and BMI values were standardized into z-scores. Linear correlations between VVV and other demographic variables were assessed. Meanwhile, we calculated odds ratios (ORs) [95% confidence level (95% Cl)] in three logistic models to assess the associations between VVV and HBP. Model 1 was unadjusted. In Model 2, age (in years, continuous) in 2018, BMI in 2018 and sex were adjusted. Based on Model 2, Model 3 additionally included SES, region, mean SBP and DBP z-scores during 2012–2017, and BP measurement times. If any of the VVV parameters was demonstrated as a significant and independent predictor of HBP, then the improvement in risk identification [represent as c-statistics, continuous net reclassification index (NRI), and integrated discrimination improvement (IDI)] of adding this specific VVV indices over established risk model (composed by variables in Model 3) was evaluated [31]. Similar statistical analyses were also conducted for HNBP. Furthermore, stratified analyses were performed to explore whether the associations between VVV and HBP were robust according to age [child (< 10 years), adolescent (≥ 10 years)] in 2018, BMI (underweight, normal, overweight, obese) in 2018, and sex (boys, girls). Two-sided *P* value < 0.05 was considered statistically significant. All statistical analyses were performed using Statistical Analysis System (SAS) software (version 9.4, SAS Institute, Cary, NC, USA).

## Results

### General characteristics of the study population

Characteristics of excluded and included subjects are present in Additional file 1: Table S1. The current study included a total of 330,618 children and adolescents who took in the HPPCA surveillance in 2018 and had annual BP measurements ≥ 3 times during 2012–2017 (Table 1). The first attendance of included participants at HPPCA covered from 2012 to 2015 (Additional file 1: Fig. S1). The enrolled subjects had a median of 5 (4–7 times) BP measurements (Additional file 1: Fig. S2). In 2018, 55,666 (16.84%) subjects were aged 9–9.9 years old, and 274,952 (83.16%) were aged 10–18 years old. Among the 42,554 individuals defined as HBP, 55.05% were boys. Of note, HBP or HNBP groups were older, had higher BMI than normotensive group (*P* < 0.0001).

**Table 1:** Characteristics of the included subjects

Variables, n (%) or mean (SD)	Total population (N = 330,618)	Normotensive (n = 252,342)	HNBP (n = 35,722)	HBP (n = 42,554)	*P* value
Age, n (%)^a^					< 0.0001
Child (9–9.9 years)	55,666 (16.84%)	39,255 (15.56%)	4841 (13.55%)	5964 (14.02%)	
Adolescent (10–18 years)	274,952 (83.16%)	213,087 (84.44%)	30,881 (86.45%)	36,590 (85.98%)	
Body mass index (kg/m^2^)^a^	19.43 ± 3.67	19.09 ± 3.45	20.17 ± 3.90	20.83 ± 4.29	
Body mass index^a,b^	0.40 ± 1.26	0.30 ± 1.23	0.64 ± 1.26	0.83 ± 1.28	< 0.0001
Sex, n (%)					< 0.0001
Boys	177,905 (53.81%)	135,069 (53.53%)	19,408 (54.33%)	23,428 (55.05%)	
Girls	152,713 (46.19%)	117,273 (46.47%)	16,314 (45.67%)	19,171 (44.95%)	
Region, n (%)					< 0.0001
Rural	166,547 (50.37%)	127,465 (50.51%)	18,169 (50.86%)	20,913 (49.14%)	
Urban	164,071 (49.63%)	124,877 (49.49%)	17,553 (49.14%)	21,641 (50.86%)	
Socioeconomic status, n (%)					< 0.0001
HSES	155,239 (46.95%)	115,793 (45.89%)	18,292 (51.21%)	21,154 (49.71%)	
LSES	175,379 (53.05%)	136,549 (54.11%)	17,430 (48.79%)	21,400 (50.29%)	
Median BP measurement times	5.19 ± 0.97	5.19 ± 0.97	5.21 ± 0.97	5.20 ± 0.97	0.341
BP at first attendance of HPPCA					
SBP (mmHg)	98.19 ± 10.12	97.73 ± 9.90	99.19 ± 10.88	100.06 ± 10.51	< 0.0001
DBP (mmHg)	62.48 ± 7.46	62.19 ± 7.36	63.17 ± 7.82	63.63 ± 7.58	< 0.0001
SBP^b^	− 0.08 ± 0.93	− 0.10 ± 0.92	0.04 ± 0.93	0.09 ± 0.96	< 0.0001
DBP^b^	0.34 ± 0.66	0.32 ± 0.66	0.41 ± 0.66	0.43 ± 0.67	< 0.0001
BP in 2018					
SBP (mmHg)	109.24 ± 13.18	105.09 ± 10.86	117.95 ± 8.98	126.71 ± 10.57	< 0.0001
DBP (mmHg)	68.20 ± 8.21	65.73 ± 6.51	74.70 ± 6.62	77.46 ± 8.79	< 0.0001
SBP^b^	0.18 ± 1.14	− 0.21 ± 0.91	0.98 ± 0.70	1.80 ± 0.83	< 0.0001
DBP^b^	0.45 ± 0.71	0.23 ± 0.55	1.02 ± 0.57	1.27 ± 0.79	< 0.0001
Mean BP during 2012–2017					
SBP (mmHg)	103.00 ± 7.82	102.09 ± 7.54	105.16 ± 7.69	106.62 ± 8.15	< 0.0001
DBP (mmHg)	65.14 ± 5.26	64.59 ± 5.13	66.54 ± 5.20	67.23 ± 5.36	< 0.0001
SBP^b^	0.11 ± 0.62	− 0.03 ± 0.57	0.41 ± 0.54	0.68 ± 0.57	< 0.0001
DBP^b^	0.41 ± 0.41	0.33 ± 0.39	0.62 ± 0.39	0.72 ± 0.42	< 0.0001
VVV during 2012–2017					
SBP-SD^c^	0.86 ± 0.39	0.82 ± 0.37	0.89 ± 0.41	1.06 ± 0.43	< 0.0001
SBP-ARV^c^	1.01 ± 0.50	0.96 ± 0.47	1.05 ± 0.50	1.30 ± 0.57	< 0.0001
SBP-CV^c^	14.27 ± 53,983.00	− 5.99 ± 61,536.74	31.62 ± 12,102.74	119.87 ± 7950.35	0.260
DBP-SD^c^	0.59 ± 0.27	0.57 ± 0.26	0.63 ± 0.27	0.70 ± 0.29	< 0.0001
DBP-ARV^c^	0.70 ± 0.35	0.67 ± 0.34	0.76 ± 0.35	0.85 ± 0.40	< 0.0001
DBP-CV^c^	129.22 ± 26,661.00	57.83 ± 17,534.74	141.27 ± 3633.13	542.44 ± 60,727.95	0.108

HBP, high blood pressure; HNBP, high normal blood pressure; SBP, systolic blood pressure; DBP, diastolic blood pressure; SD, standard deviation; ARV, actual real variability; CV, coefficient of variation; LSES, low socioeconomic status; HSES, high socioeconomic status

^a^Age and Body mass index were measured when participants took examinations in 2018

^b^Absolute BP levels and BMI values were converted into z-scores

^c^BP variability parameters were calculated based on BP z-scores during 2012–2017

Simple linear correlations of VVV with demographic (age, BMI z-score and BP measurement times) or BP z-scores are shown in Table 2. There were only weak correlations between VVV and those variables.

**Table 2:** Correlation between VVV and demographic or blood pressure variables^b^

Characteristics	SBP-SD		SBP-ARV		SBP-CV		DBP-SD		DBP-ARV		DBP-CV	
	*R2*	*P*	*R2*	*P*	*R2*	*P*	*R2*	*P*	*R2*	*P*	*R2*	*P*
Age, n (%)^a^	0.005	< 0.0001	0.002	< 0.0001	0.000	0.198	0.000	0.805	0.001	< 0.0001	0.000	0.647
Body mass index^a,b^	0.003	< 0.0001	0.003	< 0.0001	0.000	0.118	0.001	< 0.0001	0.001	< 0.0001	0.000	0.335
Median BP measurement times	0.006	< 0.0001	0.001	0.089	0.000	0.101	0.001	< 0.0001	0.000	< 0.0001	0.000	0.462
SBP at first attendance of HPPCA^b^	0.002	0.069	0.005	< 0.0001	0.000	0.945	0.004	< 0.0001	0.004	< 0.0001	0.000	0.093
DBP at first attendance of HPPCA^b^	0.002	< 0.0001	0.003	< 0.0001	0.000	0.667	0.010	< 0.0001	0.012	< 0.0001	0.000	0.590
Mean SBP during 2012–2017^b^	0.001	< 0.0001	0.002	< 0.0001	0.000	0.150	0.005	< 0.0001	0.006	< 0.0001	0.000	0.052
Mean DBP during 2012–2017^b^	0.001	0.192	0.000	0.002	0.000	0.167	0.009	< 0.0001	0.011	< 0.0001	0.000	0.774
SBP-ARV^c^	0.743	< 0.0001	–	–	–	–	–	–	–	–	–	–
SBP-CV^c^	0.000	0.046	0.000	0.478	–	–	–	–	–	–	–	–
DBP-SD^c^	0.151	< 0.0001	0.101	< 0.0001	0.000	0.543	–	–	–	–	–	–
DBP-ARV^c^	0.094	< 0.0001	0.101	< 0.0001	0.000	0.871	0.746	< 0.0001	–	–	–	–
DBP-CV^c^	0.000	0.375	0.000	0.214	0.000	0.954	0.000	0.504	0.000	0.903	–	–

VVV, visit to visit blood pressure variability; SBP, systolic blood pressure; DBP, diastolic blood pressure; SD, standard deviation; ARV, actual real variability; CV, coefficient of variation

^a^Age and body mass index were measured when participants took examinations in 2018

^b^Absolute BP levels and BMI values were converted into z-scores

^c^BP variability parameters were calculated based on BP z-scores during 2012–2017

### Association between VVV and HBP/HNBP

HBP groups had significantly greater VVV than normotensive counterparts, except for SBP-CV and DBP-CV (Table 1). Associations between VVV and HBP were explored using three logistic regression models, as shown in Table 3. Increased likelihood of HBP was observed in the crude model, yielding ORs (95% CI) of 4.40 (4.28–4.51), 3.31 (3.25–3.38), 4.88 (4.71–5.06) and 3.56 (3.47–3.66) for SBP-SD, SBP-ARV, DBP-SD and DBP-ARV, respectively. After adjusting for potential confounders in Model 3, SBP-SD (OR 5.70, 95% CI 5.54–5.87), SBP-ARV (OR 4.10, 95% CI 4.01–4.20), DBP-SD (OR 4.70, 95% CI 4.50–4.90) and DBP-ARV (OR 3.39, 95% CI 3.28–3.50) were still positively associated with risk of HBP. However, no statistically significant association was found when CV of BP was estimated. What’s more, the associations of ARV and SD of BP with probability of HBP were still obvious when restricted to younger children (< 10 years old) (Additional file 1: Table S2).

**Table 3:** Association between visit-to-visit blood pressure variability^a^ and childhood HBP

HBP	Model 1		Model 2		Model 3	
	OR (95% Cl)	*P* value	OR (95% Cl)	*P* value	OR (95% Cl)	*P* value
SBP-SD^a^	4.40 (4.28–4.51)	< 0.0001	4.15 (4.04–4.27)	< 0.0001	5.70 (5.54–5.87)	< 0.0001
SBP-ARV^a^	3.31 (3.25–3.38)	< 0.0001	3.23 (3.16–3.29)	< 0.0001	4.10 (4.01–4.20)	< 0.0001
SBP-CV^a^	1.00 (1.00–1.00)	0.671	1.00 (1.00–1.00)	0.867	1.00 (1.00–1.00)	0.857
DBP-SD^a^	4.88 (4.71–5.06)	< 0.0001	4.95 (4.77–5.13)	< 0.0001	4.70 (4.50–4.90)	< 0.0001
DBP-ARV^a^	3.56 (3.47–3.66)	< 0.0001	3.64 (3.54–3.74)	< 0.0001	3.39 (3.28–3.50)	< 0.0001
DBP-CV^a^	1.00 (1.00–1.00)	0.623	1.00 (1.00–1.00)	0.892	1.00 (1.00–1.00)	0.876

Model 1 was unadjusted. Model 2 was adjusted for age and BMI in 2018, and sex. Model 3 further included SES, region, mean SBP and DBP z-scores during 2012–2017, and BP measurement times, based on model 2

HBP, high blood pressure; DBP, diastolic blood pressure; SBP, systolic blood pressure; SD, standard deviation; ARV, actual real variability; CV, coefficient of variation

^a^BP variability parameters were calculated based on BP z-scores during 2012–2017

Consistent with the results of VVV and HBP, similar associations were found between VVV and HNBP. High SBP-SD (OR 5.20, 95% CI 5.06–5.35), SBP-ARV (OR 3.92, 95% CI 3.83–4.01), DBP-SD (OR 4.52, 95% CI 4.34–4.70) and DBP-ARV (OR 3.26, 95% CI 3.16–3.36) increased the risk of HNBP, but the association magnitudes were smaller than those of HBP (Additional file 1: Table S3).

### Sensitivity analysis

If participants with ≥ 2 BP records during 2012 to 2017 were included, the total sample size would be expanded from 330,618 to 460,306, but there was no substantial difference for the results (Additional file 1: Table S4). Consistently, the relationship between VVV, calculated using at least 2 readings during 2012–2017, with HBP among children aged < 10 years (n = 142,331) were also similar with the analyses with at least three times of BP records (Additional file 1: Table S5).

### Incremental predictive potential of VVV

Table 4 illustrates whether adding VVV to a logistic regression model consisting of mean BP values and other confounding factors could improve discriminative ability of individuals at high risk of HBP. As a whole, systolic VVVs were better risk predictors than DBP parameters. Specially, the incorporation of SBP-SD to Model 3, resulted in the highest improved predictive value for HBP (*c*-statistics increased from 0.803 to 0.847, *P* < 0.0001; NRI = 65.57%, *P* < 0.0001; IDI = 4.71%, *P* < 0.0001) among all the indices of VVV. Meanwhile, VVV also exhibited incremental predictive potential for HNBP (Additional file 1: Table S6).

**Table 4:** Reclassification and predictive potential value of VVV for childhood HBP

HBP	C-statistics		Continuous NRI, %		IDI, %	
	Estimate (95% Cl)	*P* value	Estimate	*P* value	Estimate	*P* value
Model 3	0.803 (0.798–0.805)		Reference		Reference	
Model 3 + SBP-SD^a^	0.847 (0.842–0.853)	< 0.0001	65.57%	< 0.0001	4.71%	< 0.0001
Model 3 + SBP-ARV^a^	0.848 (0.843–0.853)	< 0.0001	62.69%	< 0.0001	5.59%	0.003
Model 3 + DBP-SD^a^	0.822 (0.818–0.826)	< 0.0001	38.02%	< 0.0001	1.51%	< 0.0001
Model 3 + DBP-ARV^a^	0.822 (0.818–0.826)	< 0.0001	37.56%	< 0.0001	1.86%	0.003

Model 3 included age and BMI in 2018, sex, SES, region, mean SBP and DBP z-scores during 2012–2017, and BP measurement times

HBP, high blood pressure; DBP, diastolic blood pressure; SBP, systolic blood pressure; SD, standard deviation; ARV, actual real variability; CV, coefficient of variation; NRI, net reclassification index; IDI, integrated discrimination improvement

^a^BP variability parameters were calculated based on BP z-scores during 2012–2017

### Stratified analyses

**Fig. 2:**
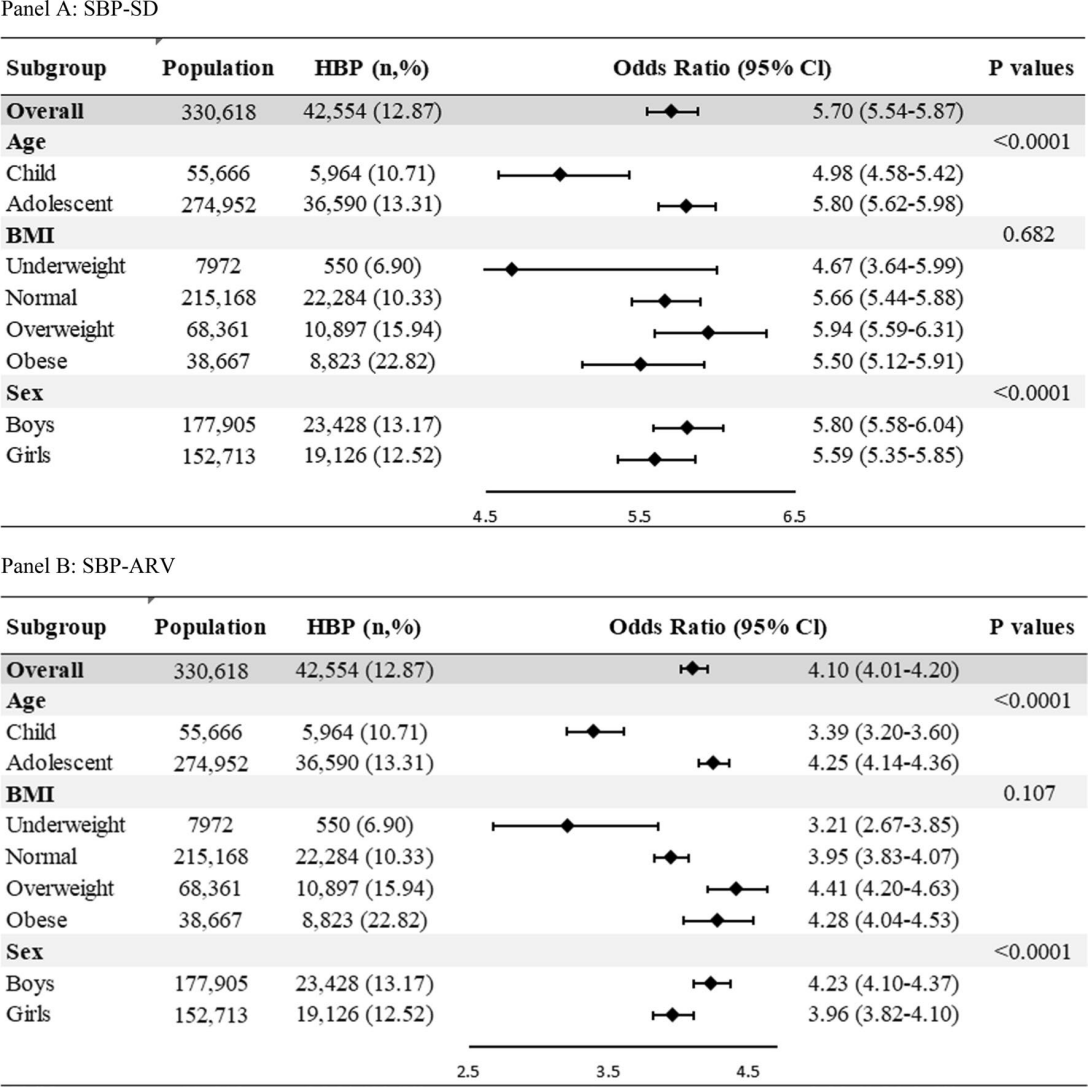
Stratified analysis for associations between systolic visit-to-visit blood pressure variability and childhood HBP (**a** SBP-SD; **b** SBP-ARV)

To determine whether associations between SBP-SD or SBP-ARV and HBP were modified by some confounding factors, we further performed stratified analyses according to age, sex and BMI status in 2018. The association between systolic VVV and HBP was stronger in boys, after adjusting for variables in Model 3 (Fig. 2).

## Discussion

In the current study, we found that SD or ARV of BP was significantly and independently associated with HBP in children and adolescents, beyond the average BP values. In addition, systolic VVV, especially SBP-SD, could potentially become a variable to aid in the discrimination of participants at high risk of HBP.

VVV was significantly higher in individuals with HBP than its counterparts in the current study, although another study revealed that VVV was not significantly different between four normotensive and 20 hypertensive children and adolescents with renal disease in Japan [32]. Difference in sample size, ethnicity and disease setting may contribute to the discrepancy. The physiopathological mechanisms linking high VVV to HBP remains obscure. Under normal physiological conditions, BPV mainly represents a response to environmental stimulation and challenges of daily life, which aimed at maintaining the so-called BP “homeostasis” [8]. Sustained elevations in BPV may also reflect alterations in the mechanisms responsible for cardiovascular homeostasis or underlying pathological conditions, and may therefore represent a source of damage to the cardiovascular system. For example, VVV was found to be associated with markers of cardiac, vascular, and cerebral diseases as well as albuminuria [8]. Furthermore, VVV has been related with central sympathetic overactivity, variations in renin–angiotensin–aldosterone system stimulation, adverse alterations in cardiac structure or function (i.e., left ventricular dysfunction, atrial fibrillation), as well as vascular target organ damage (i.e., carotid atherosclerosis and stiffness) in adults [12, 33] and the elderly [34]. In pediatrics with renal disease, increased VVV was tied with reduced eGFR [32]. Thus greater VVV may represent an inability to maintain hemodynamic homeostasis, and contribute to HBP in children and adolescents.

Interestingly, systolic VVV showed stronger associations with HBP than diastolic VVV in the current study. In adults, regarding prediction of cardiovascular endpoints, SBP was considered to be better than DBP [35], and systolic VVV was superior to diastolic VVV [36]. Meanwhile, it was reported that systolic BPV was higher than diastolic BPV in adolescents with essential hypertension [37]. Stabouli et al*.* also showed that increased SBP variability was closely associated with arterial stiffness (assessed by pulse wave velocity) in children and adolescents [18]. One possible reason for this finding might be owing to different hemodynamic mechanisms of BP increase [38]. Pediatric elevated SBP was associated with higher urinary noradrenaline levels, suggesting hyperactivity of the sympathetic nervous system (SNS) [39], while no such phenomenon was found for elevated DBP [40]. Moreover, in the conscious rats experiment, systolic BPV levels reflected α-adrenoceptor-mediated vasomotor function and β-adrenoceptor-mediated cardiac sympathetic function [41]. The activation of the SNS and cardiac sympathetic function was closely associated with left ventricular systolic function [42], therefore, hyperactivity of the sympathetic function could potentially induce a deeper impairment of systolic function, high SBP variability and subsequent HBP.

We also found that associations between VVV and HBP were enhanced when we restricted to boys. Boys are more susceptible to the impact of VVV than girls, which could possibly be explained by the varied timing and duration of sexual maturation between boys and girls [43]. Differences in lifestyles (including diet habits, physical activities, etc.) may also account for the gender-difference in the associations of VVV with HBP, so the potential gender-specific associations need to be elucidated further.

The present study found stronger links between SD or ARV of BP and childhood HBP, instead of CV. It seemed that SD or ARV, as indicators of BP variability, were more widely assessed in the field of pediatrics [16, 22, 23, 32, 44], as only a few studies have evaluated CV [16, 44]. Our results also were in agreement with previous studies in adults. Chen et al. demonstrated that even after adjusting for mean childhood BP levels, adult hypertension was associated with increased BPV in terms of SD of serial BP measurements obtained during childhood [44]. It was reported that both high BP-SD and BP-CV might increase the risk of chronic kidney disease, but the former association was more significant [45]. One meta-analysis provided evidence that great SBP-SD may indicate an increased risk of stroke (RR 1.20, 95% CI 1.07–1.35); however, when CV was considered, the hazard for stroke was not stable (RR 1.12, 95% CI 1.00–1.26) [46]. In addition, Mokadem et al. indicated that ARV was the only independent predictor of target organ damage in contrast to SD and CV [47]. Further studies are warrant to determine the most appropriate index for the evaluation of BP variability.

The current study benefits from its large sample size and representativeness of the general pediatric population that referred for hypertension evaluation. Almost all children and adolescents of considered grades would be recruited to attend HPPCA, which may contribute to reduced selection bias of the current study. Besides, observational studies have suggested that VVV may be importantly influenced by seasonal changes in weather conditions [48]. But in HPPCA [24], all the participants were regularly measured at a settled time within a year. However, several limitations should be admitted. First, in this study, we just used the average of three separate BPs at single visit in 2018 to determine HBP or HNBP status. Ideally, hypertension is suggested to be assessed based on BP values from at least three separate office visits [29]. Due to limited time and energy, BP was only measured at single time point among the hundreds of thousands of child/adolescent in HPPCA, and it was also difficult to track those with abnormal BP values during the HPPCA surveillance to further determine their status of hypertension. As a result, we used terms “high BP” and “high normal BP” instead of “hypertension” and “prehypertension”, and our findings may be subject to BP misclassification. Nevertheless, this systematic overestimation is unlikely to bias our trend results [24]. Second, the participants were only recruited from Suzhou, a developed city in Eastern China. Besides, the vast majority of the included subjects were adolescents. Therefore, our findings may not be applicable to children with age < 10 years or generalizable to other racial/ethnic populations. Third, some potential confounding parameters that may influence the development of HBP, such as living environment, dietary pattern, and physical activity, were not available in the current study. Fourth, the relationship between VVV and HBP may not necessarily be causal, given the observational nature of the study. Last but not least, BP variability may be independently involved in the progression and severity of cardiac, cerebral and renal target-organ damage. For example, greater BPV appears to increase the risk of adverse alterations in cardiac structure in both adults and pediatrics independent of mean BP levels [12, 16]. However, such information is not available for the current study, preventing us to effectively detect the independent prognostic value of VVV. Further studies focus on VVV and other target-organ damage are encouraged.

Our findings support the view that cardiovascular risk may depend not only on average BP levels but also on the degree of BPV in both children and adolescents. The clinical relevance of evaluating the dynamic patterns of several cardiovascular and metabolic variables, including BPV overtime, in addition to the assessment of magnitude and duration of average BP level elevation, has been emphasized in a large number of studies. VVV is a non-invasive, inexpensive and applicable method; therefore, the application of VVV for minoring childhood HBP should be considered, especially for similar kinds of school-based surveillance. Besides, the current study indicated that more attention should be paid for boys, who may be more vulnerable to higher VVV.

## Conclusion

In conclusion, the present study suggested that VVV increased in children and adolescents with HBP in China. In addition, systolic VVV, especially SBP-SD, could be potentially helpful for detecting HBP on the basis of other risk variables. The relationship and mechanisms between VVV and HBP hazard during childhood and adolescence need to be further investigated.

## Supplementary Information

**Additional file 1: Table S1**. Differences between characteristics of included and excluded individuals. **Table S2**. Association between visit-to-visit blood pressure variability* and childhood HBP among those younger children (<10 years old, n = 55,666). **Table S3**. Association between visit-to-visit blood pressure variability* and childhood HNBP. **Table S4**. Association between visit-to-visit blood pressure variability* and childhood HBP among those with at least 2 readings during 2012-2017 (n = 460,306). **Table S5**. Association between visit-to-visit blood pressure variability* and childhood HBP among those younger children (<10 years old) with at least 2 readings during 2012-2017 (n = 142,331). **Table S6**. Reclassification and predictive potential value of VVV for childhood HNBP. **Fig. S1**. First attendance year of each included participant at HPPCA. **Fig. S2**. BP measurement times of the enrolled participants during 2012–2017.

## Data Availability

The datasets used and/or analysed during the current study are available from the corresponding author on reasonable request.

## Abbreviations

VVV: Visit-to-visit blood pressure variability
HBP: High blood pressure
SBP: Systolic blood pressure
DBP: Diastolic blood pressure
SD: Standard deviation
CV: Coefficient variation
ARV: Average real variability
