# Supplementary material for: School-based surveillance on visit-to-visit blood pressure variability and high blood pressure in children and adolescents
Source: BMC Cardiovasc Disord. 2021 Mar 17;21:141. doi: 10.1186/s12872-021-01947-1 (PMC7967982; doi:10.1186/s12872-021-01947-1)
Supplement: Supplementary file 1 — Additional file 1: Table S1. Differences between characteristics of included and excluded individuals. Table S2. Association between visit-to-visit blood pressure variability* and childhood HBP among those younger children (<10 years old, n = 55,666). Table S3. Association between visit-to-visit blood pressure variability* and childhood HNBP. Table S4. Association between visit-to-visit blood pressure variability* and childhood HBP among those with at least 2 readings during 2012-2017 (n = 460,306). Table S5. Association between visit-to-visit blood pressure variability* and childhood HBP among those younger children (<10 years old) with at least 2 readings during 2012-2017 (n = 142,331). Table S6. Reclassification and predictive potential value of VVV for childhood HNBP. Fig. S1. First attendance year of each included participant at HPPCA. Fig. S2. BP measurement times of the enrolled participants during 2012–2017. [file 12872_2021_1947_MOESM1_ESM.docx]

**Supplementary File**

**School-based surveillance on visit-to-visit blood pressure variability and high blood pressure in children and adolescents**

Jiaxiang Wang, MD^a#^, Hui Shen, MD, PhD^b#^, Jieyu Liu, MD^a^, Chengqi Xiao, MD^a^, Cailong Chen, MD, PhD^c^, Haoyue Teng, MD^a^, Jia Hu, MD, PhD^b*^, Jieyun Yin, MD, PhD^a*^

#These authors contributed equally to this work.

^a^ Jiangsu Key Laboratory of Preventive and Translational Medicine for Geriatric Diseases, School of Public Health, Medical College of Soochow University, Suzhou, Jiangsu, China;

^b^ Suzhou Center for Disease Prevention and Control, Suzhou, Jiangsu, China;

^c^ Children health management center, Children's Hospital of Soochow University, Suzhou, Jiangsu, China.

**Correspondence to:**

Jia Hu, MD, PhD, Suzhou Center for Disease Prevention and Control, 72 Sanxiang Road, Suzhou, Jiangsu 215004, China. Tel./fax: +86-512-62701715. E-mail address: hujia200606@163.com.

Jieyun Yin, MD, PhD, School of Public Health, Medical College of Soochow University, 199 Renai Road, Suzhou, Jiangsu, China 215123. Tel./fax: +86 0512 6588036 / Email address: [jyyin@suda.edu.cn](mailto:jyyin@suda.edu.cn)

**Supplementary Figure**

**Supplementary Figure 1:** First attendance year of each included participant at HPPCA.





**Supplementary Figure 2:** BP measurement times of the enrolled participants during 2012-2017.





| **Supplementary Table 1 Differences between characteristics of included and excluded individuals.** | | | |
| --- | --- | --- | --- |
| Variables, n (%) or mean (SD) | **Included**  **N=330,618** | **Excluded N=345,084** | **P-value** |
| Age, n (%)^#^ |  |  | <0.0001 |
| Child (9-9.9 years) | 55666 (16.84%) | 235545 (68.26%) |  |
| Adolescent (10-18 years) | 274952 (83.16%) | 109539 (31.74%) |  |
| Body mass index (kg/m^2^)^#^ | 19.43±3.67 | 19.48±3.93 | <0.0001 |
| Body mass index^#^* | 0.40±1.26 | 0.41±1.28 | <0.0001 |
| Sex |  |  | 0.707 |
| Boys (%) | 177905 (53.81%) | 185250 (53.68%) |  |
| Girls (%) | 152713 (46.19%) | 159834 (46.32%) |  |
| Region |  |  | <0.0001 |
| Rural, n (%) | 166547 (50.37%) | 184810 (53.56%) |  |
| Urban, n (%) | 164071 (49.63%) | 160274 (46.44%) |  |
| Socioeconomic status, n (%) |  |  | <0.0001 |
| HSES, n (%) | 155239 (46.95%) | 161152 (46.70%) |  |
| LSES, n (%) | 175379 (53.05%) | 183932 (53.30%) |  |
| Median BP measurement times | 5.19±0.97 | 2.44±0.50 | <0.0001 |
| BP at first attendance of HPPCA |  |  |  |
| SBP (mmHg) | 98.19±10.12 | 103.01±12.18 | <0.0001 |
| DBP (mmHg) | 62.48±7.46 | 76.52±7.92 | <0.0001 |
| SBP* | -0.08±0.93 | -0.12±1.10 | <0.0001 |
| DBP* | 0.34±0.66 | 0.47±0.79 | <0.0001 |
| BP in 2018 |  |  |  |
| SBP (mmHg) | 109.24±13.18 | 117.82±13.10 | <0.0001 |
| DBP (mmHg) | 68.20±8.21 | 75.52±8.32 | <0.0001 |
| SBP* | 0.18±1.14 | 0.25±1.10 | <0.0001 |
| DBP* | 0.45±0.71 | 0.57±0.79 | <0.0001 |
| Mean BP during 2012-2017 |  |  |  |
| SBP (mmHg) | 103.00±7.82 | 117.02±9.63 | <0.0001 |
| DBP (mmHg) | 65.14±5.26 | 72.43±8.12 | <0.0001 |
| SBP* | 0.11±0.62 | 0.24±0.99 | <0.0001 |
| DBP* | 0.41±0.41 | 0.56±0.71 | <0.0001 |

SBP, systolic blood pressure; DBP, diastolic blood pressure; SD, standard deviation; ARV, actual real variability; CV, coefficient of variation; LSES, low socioeconomic status; HSES, high socioeconomic status.

^#^Age and Body mass index were measured when participants took examinations in 2018.

* Absolute BP levels and BMI values were converted into z-scores.

| **Supplementary Table 2. Association between visit-to-visit blood pressure variability* and childhood HBP among those younger children (<10 years old, n=55,666).** | | | | | | | | |
| --- | --- | --- | --- | --- | --- | --- | --- | --- |
| HBP | Model 1 | |  | Model 2 | |  | Model 3 | |
|  | OR (95% Cl) | P-value |  | OR (95% Cl) | P-value |  | OR (95% Cl) | P-value |
| SBP-SD* | 5.50 (5.17-5.86) | <0.0001 |  | 5.38 (5.05-5.73) | <0.0001 |  | 5.53 (5.14-5.94) | <0.0001 |
| SBP-ARV* | 3.39 (3.24-3.54) | <0.0001 |  | 3.35 (3.20-3.50) | <0.0001 |  | 3.65 (3.47-3.84) | <0.0001 |
| SBP-CV* | 1.00 (1.00-1.00) | 0.643 |  | 1.00 (1.00-1.00) | 0.975 |  | 1.00 (1.00-1.00) | 0.983 |
| DBP-SD* | 5.94 (5.48-6.44) | <0.0001 |  | 5.98 (5.51-6.49) | <0.0001 |  | 4.68 (4.27-5.13) | <0.0001 |
| DBP-ARV* | 3.81 (3.60-4.04) | <0.0001 |  | 3.83 (3.61-4.06) | <0.0001 |  | 3.52 (3.30-3.76) | <0.0001 |
| DBP-CV* | 1.00 (1.00-1.00) | 0.632 |  | 1.00 (1.00-1.00) | 0.912 |  | 1.00 (1.00-1.00) | 0.978 |

HBP: high blood pressure; DBP, diastolic blood pressure; SBP, systolic blood pressure; SD, standard deviation; ARV, actual real variability; CV, coefficient of variation.
*BP variability parameters were calculated based on BP z-scores during 2012-2017.

Model 1 was unadjusted.
Model 2 was adjusted for age and BMI in 2018, and sex.
Model 3 further included SES, region, mean SBP and DBP z-scores during 2012-2017, and BP measurement times, based on model 2.

| **Supplementary Table 3. Association between visit-to-visit blood pressure variability* and childhood HNBP.** | | | | | | | | |
| --- | --- | --- | --- | --- | --- | --- | --- | --- |
| HNBP | Model 1 | |  | Model 2 | |  | Model 3 | |
|  | OR (95% Cl) | P-value |  | OR (95% Cl) | P-value |  | OR (95% Cl) | P-value |
| SBP-SD* | 3.93 (3.82-4.03) | <0.0001 |  | 3.72 (3.63-3.82) | <0.0001 |  | 5.20 (5.06-5.35) | <0.0001 |
| SBP-ARV* | 3.11 (3.05-3.17) | <0.0001 |  | 3.03 (2.97-3.09) | <0.0001 |  | 3.92 (3.83-4.01) | <0.0001 |
| SBP-CV* | 1.00 (1.00-1.00) | 0.641 |  | 1.00 (1.00-1.00) | 0.817 |  | 1.00 (1.00-1.00) | 0.865 |
| DBP-SD* | 4.26 (4.11-4.41) | <0.0001 |  | 4.29 (4.14-4.45) | <0.0001 |  | 4.52 (4.34-4.70) | <0.0001 |
| DBP-ARV* | 3.24 (3.16-3.33) | <0.0001 |  | 3.29 (3.21-3.38) | <0.0001 |  | 3.26 (3.16-3.36) | <0.0001 |
| DBP-CV* | 1.00 (1.00-1.00) | 0.589 |  | 1.00 (1.00-1.00) | 0.886 |  | 1.00 (1.00-1.00) | 0.893 |

HNBP: high normal blood pressure; DBP, diastolic blood pressure; SBP, systolic blood pressure; SD, standard deviation; ARV, actual real variability; CV, coefficient of variation.
*BP variability parameters were calculated based on BP z-scores during 2012-2017.

Model 1 was unadjusted.
Model 2 was adjusted for age and BMI in 2018, and sex.
Model 3 further included SES, region, mean SBP and DBP z-scores during 2012-2017, and BP measurement times, based on model 2.

| **Supplementary Table 4. Association between visit-to-visit blood pressure variability* and childhood HBP among those with at least 2 readings during 2012-2017 (n=460,306).** | | | | | | | | |
| --- | --- | --- | --- | --- | --- | --- | --- | --- |
| HBP | Model 1 | |  | Model 2 | |  | Model 3 | |
|  | OR (95% Cl) | P-value |  | OR (95% Cl) | P-value |  | OR (95% Cl) | P-value |
| SBP-SD* | 3.53 (3.46-3.61) | <0.0001 |  | 3.42 (3.35-3.49) | <0.0001 |  | 5.53 (5.41-5.65) | <0.0001 |
| SBP-ARV* | 2.84 (2.80-2.88) | <0.0001 |  | 2.80 (2.76-2.84) | <0.0001 |  | 3.97 (3.90-4.04) | <0.0001 |
| SBP-CV* | 1.00 (1.00-1.00) | 0.631 |  | 1.00 (1.00-1.00) | 0.873 |  | 1.00 (1.00-1.00) | 0.889 |
| DBP-SD* | 4.09 (3.98-4.20) | <0.0001 |  | 4.12 (4.00-4.23) | <0.0001 |  | 5.14 (4.98-5.31) | <0.0001 |
| DBP-ARV* | 3.17 (3.10-3.23) | <0.0001 |  | 3.22 (3.16-3.29) | <0.0001 |  | 3.62 (3.53-3.70) | <0.0001 |
| DBP-CV* | 1.00 (1.00-1.00) | 0.593 |  | 1.00 (1.00-1.00) | 0.803 |  | 1.00 (1.00-1.00) | 0.826 |

HBP: high blood pressure; DBP, diastolic blood pressure; SBP, systolic blood pressure; SD, standard deviation; ARV, actual real variability; CV, coefficient of variation.
*BP variability parameters were calculated based on BP z-scores during 2012-2017.

Model 1 was unadjusted.
Model 2 was adjusted for age and BMI in 2018, and sex.
Model 3 further included SES, region, mean SBP and DBP z-scores during 2012-2017, and BP measurement times, based on model 2.

| **Supplementary Table 5. Association between visit-to-visit blood pressure variability* and childhood HBP among those younger children (<10 years old) with at least 2 readings during 2012-2017 (n=142,331).** | | | | | | | | |
| --- | --- | --- | --- | --- | --- | --- | --- | --- |
| HBP | Model 1 | |  | Model 2 | |  | Model 3 | |
|  | OR (95% Cl) | P-value |  | OR (95% Cl) | P-value |  | OR (95% Cl) | P-value |
| SBP-SD* | 4.92 (4.75-5.10) | <0.0001 |  | 4.88 (4.71-5.06) | <0.0001 |  | 6.00 (5.75-6.26) | <0.0001 |
| SBP-ARV* | 3.19 (3.11-3.26) | <0.0001 |  | 3.18 (3.10-3.26) | <0.0001 |  | 3.95 (3.83-4.07) | <0.0001 |
| SBP-CV* | 1.00 (1.00-1.00) | 0.672 |  | 1.00 (1.00-1.00) | 0.885 |  | 1.00 (1.00-1.00) | 0.864 |
| DBP-SD* | 5.78 (5.52-6.05) | <0.0001 |  | 5.84 (5.57-6.11) | <0.0001 |  | 5.59 (5.29-5.89) | <0.0001 |
| DBP-ARV* | 3.84 (3.72-3.96) | <0.0001 |  | 3.87 (3.75-3.99) | <0.0001 |  | 4.10 (3.95-4.26) | <0.0001 |
| DBP-CV* | 1.00 (1.00-1.00) | 0.587 |  | 1.00 (1.00-1.00) | 0.815 |  | 1.00 (1.00-1.00) | 0.842 |

HBP: high blood pressure; DBP, diastolic blood pressure; SBP, systolic blood pressure; SD, standard deviation; ARV, actual real variability; CV, coefficient of variation.
*BP variability parameters were calculated based on BP z-scores during 2012-2017.

Model 1 was unadjusted.
Model 2 was adjusted for age and BMI in 2018, and sex.
Model 3 further included SES, region, mean SBP and DBP z-scores during 2012-2017, and BP measurement times, based on model 2.

| **Supplementary Table 6. Reclassification and predictive potential value of VVV for childhood HNBP.** | | | | | | |
| --- | --- | --- | --- | --- | --- | --- |
| HNBP | C-statistics | | Continuous NRI,% | | IDI,% | |
|  | Estimate (95% Cl) | P-value | Estimate | P-value | Estimate | P-value |
| Model 3 | 0.741 (0.738-0.744) |  | Reference |  | Reference |  |
| Model 3+SBP-SD* | 0.751 (0.748-0.753) | <0.0001 | 58.43% | <0.0001 | 3.28% | <0.0001 |
| Model 3+SBP-ARV* | 0.752 (0.749-0.754) | <0.0001 | 55.60% | <0.0001 | 4.16% | 0.006 |
| Model 3+DBP-SD* | 0.748 (0.746-0.751) | <0.0001 | 32.74% | <0.0001 | 1.21% | <0.0001 |
| Model 3+DBP-ARV* | 0.749 (0.747-0.752) | <0.0001 | 30.96% | <0.0001 | 1.76% | 0.004 |

HNBP: high normal blood pressure; DBP, diastolic blood pressure; SBP, systolic blood pressure; SD, standard deviation; ARV, actual real variability; CV, coefficient of variation; NRI: net reclassification index; IDI: integrated discrimination improvement.
*BP variability parameters were calculated based on BP z-scores during 2012-2017.

Model 3 included age and BMI in 2018, sex, SES, region, mean SBP and DBP z-scores during 2012-2017, and BP measurement times.
